# Pilot Study: Heart Rate and Heart Rate Variability Indices in Mules Evaluated by 24-Hour Electrocardiogram

**DOI:** 10.3390/ani15162438

**Published:** 2025-08-20

**Authors:** Lauren T. Maas, Jessica M. Morgan, Jordan Case, David D. Chell, Amy K. McLean

**Affiliations:** 1 Department of Medicine and Epidemiology, School of Veterinary Medicine, University of California, Davis, CA 95616, USA; ltmaas@ucdavis.edu (L.T.M.); jmmorgan@ucdavis.edu (J.M.M.); jmcase@ucdavis.edu (J.C.); 2 Department of Animal Science, University of California, Davis, CA 95616, USA; ddchell@ucdavis.edu

**Keywords:** cardiac, ECG, EKG, Holter monitor, arrhythmia, equine

## Abstract

Mules and donkeys are the 5th largest equine population in the U.S., yet basic information such as heart rate and arrhythmia prevalence in mules has not been described. Analysis of heart rate variability (HRV) provides valuable insight into physiologic and autonomic differences between mules, horses, and donkeys. We aimed to pilot the evaluation of heart rate and variability in a healthy population of mules using a 24 h electrocardiogram. Twenty-four-hour ambulatory electrocardiogram (aECG) recordings were collected from an athletic population of seven privately owned healthy mules in Northern California. Vision 5 Holter Analysis Software was utilized for ECG review and analysis. An average heart rate of 32–42 bpm, with a mean of 36 ± 3 bpm for 23.8 ± 0.2 h, was recorded. Minimum heart rate was 16–24 bpm, and maximum was 70–156 bpm. Individual mules had supraventricular complexes, sinoatrial heart block, and second-degree atrioventricular block during the observation period. Electrocardiographic evaluation of mules over 24 h found heart rate (HR) to be similar to horses and lower than in donkeys. Arrhythmias were consistent with the range of cardiac rhythms observed in horses and more frequent than reported in donkeys. Further investigation with a larger sample size of mules is warranted to determine if reference values for horses should continue to be used to assess mules and to investigate the prevalence of arrhythmias and their clinical relevance in the mule.

## Simple Summary

Information on mule heart rate, variability, and rhythm irregularities, such as arrhythmia prevalence, is lacking in the scientific literature. Historically, due to mules and donkeys being members of the Equidae family, assumptions have been made that they are physiologically the same as horses. However, there is evidence of physiological differences between horses and donkeys that have important clinical consequences. Therefore, there is reason to evaluate whether mules, the result of a cross between a female horse (mare) and a male donkey (jack), may differ physiologically from horses as well. Piloting the use of 24 h electrocardiograms (ECG) was feasible and provided valuable data suggesting 24 h ECGs may be valuable for future evaluation of cardiac rhythm and clinical arrhythmias in mules. Data from this pilot suggests heart rate (bpm) and HRV were closer to those of horses than donkeys.

## 1. Introduction

Mules and donkeys are the 5th largest equid population in the United States, with 21% of equine operations in the United States reporting to have a mule or donkey [1,2]. Globally, mules are an important equid hybrid that have traditionally been under investigated [3]. Heart rate (HR) and heart rate variability (HRV) are important clinical parameters that allow for monitoring of health, disease status, and stress levels in animals. HR measures the number of heart beats per minute (bpm), with elevations often indicating pain, stress, and/or disease. HRV measures the variation in individual beat-to-beat intervals and can serve as a surrogate for autonomic tone, making it a useful measure of stress. While mules are essential working equids globally, their popularity as recreational and competition equids in the United States have led to an increase in clinical cases, and there is a need for more baseline information on mule physiology. Existing research on HR and HRV in mules is lacking, limiting the use of these tools for evaluating mule health and welfare.

While the mule is a hybrid of the horse and donkey, both members of the Equidae family, there are physiological differences between horses and donkeys that have important clinical implications [4]. It is a disservice to mules to assume they are physiologically the same as horses or donkeys, and this common assumption can result in misinterpretation of their health status. The accepted resting HR of horses ranges from 28 to 44 bpm [5,6,7], with the resting HR of donkeys being reported slightly higher, ranging from 36 to 65 bpm, depending on the source [8,9,10,11]. Early investigation of the vital signs of mules reported a mean pulse rate between 29 and 37 bpm based on palpation of the external maxillary artery [12]. Another report found a population of Iberian mules to have an HR of 43 bpm, determined via auscultation [13]. Measurements of mule heart rate over extended periods based on electrocardiograms (ECGs) may allow for future description of normal diurnal variation in HR and rhythm that occurs within each day, without the physiologic implications of handling. Previous studies in donkeys have highlighted species differences in ECG data compared to horses, reinforcing the need for data in mules [13,14].

In addition to reporting HR, a continuous ECG of mules allows for calculation of HRV parameters, which are often used as a surrogate measure of autonomic tone, stress level, and evaluation of the cardiac rhythm. HRV has been used extensively in the behavioral evaluation of horses and is rarely reported in donkeys. To the authors’ knowledge, no data on HRV is available in mules. This information will provide valuable insights for future studies aiming to evaluate stress levels and ultimately improve mule welfare. Evaluation of the cardiac rhythm will allow for a better understanding of the normal electrocardiogram of mules and provide important baseline information for the evaluation of clinical arrhythmias.

The primary objective of this study is to preliminarily describe HR and HRV parameters in a clinically healthy population of mules utilizing 24 h ECG monitoring. By successfully reporting HR and HRV values specific to mules over 24 h, this study contributes valuable data that can inform future research and clinical practice, promoting a more complete understanding of mule physiology and supporting the advancement of their welfare.

## 2. Materials and Methods

### 2.1. Animals

Twenty-four-hour ambulatory electrocardiogram (aECG) recordings were collected from 7 privately owned mules (Table 1) residing in Northern California. All mules were sired by Mammoth jacks. Mules were housed individually for an overnight acclimation period prior to the beginning of their ECG recording. Mules remained individually housed for the duration of their recording and were maintained on their routine diet of grass and/or alfalfa hay morning (approximately 0700–0730) and evening (approximately 1800–1830) and had water available ad libitum. One mule was housed at a separate facility from the other six and was recorded two weeks prior to the others as a pilot study; however, routine management, as well as management for the duration of the study, of all mules was the same. Physical examinations were performed prior to the placement of the aECGs. All mule owners provided written consent prior to their animals’ participation in the study, which was conducted in accordance with the University of California, Davis Institutional Animal Care and Use guidelines for field studies that do not require a proposal number.

### 2.2. aECG Recordings

**Figure 1:**
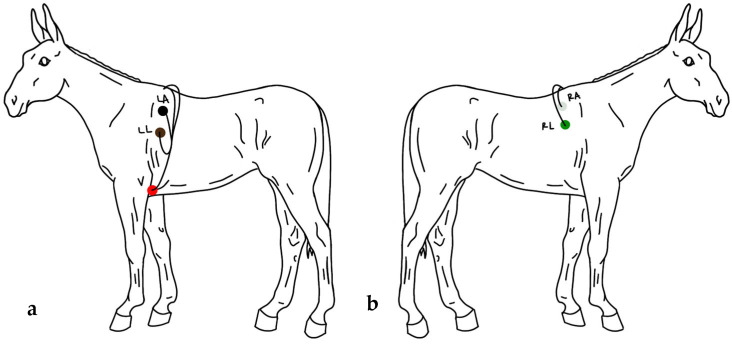
Illustration of placement of electrodes. (**a**) On the left side of the mule, one electrode (V) was placed caudal to the xiphoid process on the ventral midline. One electrode (LL) was placed 50 cm down from the dorsal midline, caudal to the long head of the triceps. One electrode (LA) was placed 20 cm down from the dorsal midline, caudal to the scapular cartilage. (**b**) On the right side of the mule, one electrode (RA) was placed 20 cm down from the dorsal midline, caudal to the scapular cartilage. The final electrode (RL) was placed 40 cm down from the dorsal midline.

A 5-lead aECG was recorded utilizing a Burdick 48 Hr H3+ Holter Recorder (Hill-Rom Holdings, Inc., Chicago, IL, USA) with 3 separate channels. Hair was clipped in approximately 2” × 2” squares and cleaned with 70% isopropyl alcohol at electrode sites. Skintact W-601 Solid Gel Cloth Electrodes (Leonhard Lang USA, Inc., Inverness, FL, USA) were placed with three on the left side and two on the right in a modified base apex configuration. On the left, one electrode was placed caudal to the xiphoid process on the ventral midline, one 50 cm down from the dorsal midline caudal to the long head of the triceps, and one 20 cm down from the dorsal midline caudal to the scapular cartilage. On the right side of the mule, one electrode was placed 20 cm down from the dorsal midline, caudal to the scapular cartilage, and the second electrode on the right was placed 40 cm down from the dorsal midline (Figure 1). Electrodes were secured with super glue (The Gorilla Glue Company, Cincinnati, OH, USA) as necessary. Elastikon Elastic Tape (Johnson & Johnson, New Brunswick, NJ, USA) was then placed around the heart girth, followed by an adjustable elastic surcingle to hold the aECG monitor, leads, and electrodes in place.

Vision 5 Holter Analysis Software (Welch Allyn, Inc. Skaneateles Falls, NY, USA) was utilized for ECG review and analysis. ECGs were reviewed by two authors (DC and LM), and erroneous complex identifications, such as misidentification of P waves, T waves, or artifacts as QRS complexes, were corrected prior to secondary review of the ECGs by a third author (JM). All authors performing the review were blinded to mule identification. The software parameters were set to identify supraventricular premature complexes with deviation >20% from previous R-R intervals, atrial fibrillation, ventricular complexes, and pauses defined as 3000 msec or greater. Sinus block and second-degree atrioventricular block (2DAVB) were both classified as pauses, as they were both considered to be physiologic, and the available software package did not allow for repeatable differentiation. The R-R interval deviation was set to a maximum of 20% based on resting recommendations in the existing equine literature [14,15,16]. Supraventricular premature complexes (SVPCs) were characterized as premature atrial depolarization with >20% shorter deviation than the previous beat. Overall deviation was evaluated subjectively during an aECG review, and periods of sinus arrhythmia of >20% deviation in R-R interval were manually removed from the SVPC category. Ventricular complexes (VCs) were characterized as ventricular depolarization marked by the absence of detectable P-waves and a change in QRS morphology. Total recording duration, total analyzed recording duration, minimum and maximum HR, average HR, number of SVPCs, VCs, and pauses (2DAVB or sinus block), hourly mean HR, and hourly pauses were recorded. Time-dependent HRV measures, including standard deviation of normal intervals (SDNN), SDNN Index, standard deviation of the average NN intervals (SDANN), root mean square of successive intervals (RMSSD), and Triangular index (TI), were recorded for the duration of the recording. Additionally, RMSSD and SDNN were recorded hourly from the start of the recording.

### 2.3. Statistical Analysis

Statistical analysis was performed utilizing GraphPad Prism 10 (GraphPad Software, Boston, MA, USA). Descriptive statistics were used for all parameters. Normality was assessed by a Shapiro–Wilk test. Normally distributed data are reported as mean ± standard deviation (SD), and nonparametric data as median, interquartile range (IQR), and minimum-maximum.

## 3. Results

### 3.1. Auscultation Findings

On auscultation, one mule (mule 1) was noted to have 2DAVB. No other arrhythmias or murmurs were auscultated. Resting HR was recorded at the time of auscultation for each mule. Heart rate as determined by auscultation ranged from 28 to 40 bpm with a mean ± SD of 34 ± 5 bpm.

### 3.2. aECG Results

**Figure 2:**
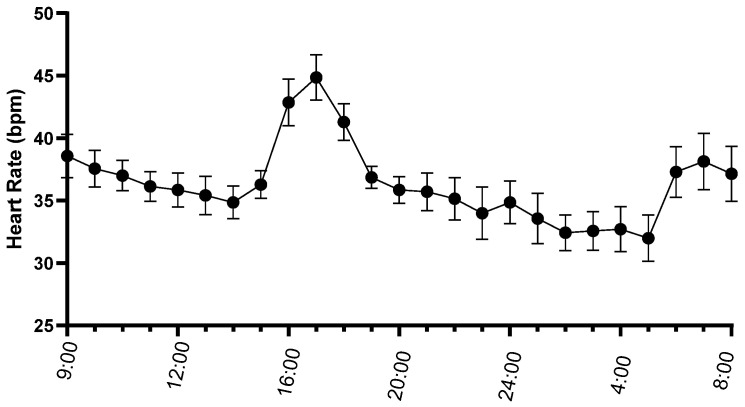
Mean ± standard error of the mean of heart rate (beats/min) of seven mules during 24 h ambulatory electrocardiograms.

Diagnostic quality ECG recordings with a mean recording duration of 23.8 ± 0.2 h and an analyzed duration of 23.7 ± 0.3 h were obtained in all seven mules. Average heart rate over the recording ranged from 32 to 42 bpm with a mean of 36 ± 3 bpm (Figure 2). Minimum heart rate ranged from 16 to 24 bpm with a mean of 20 ± 3 bpm. Maximum heart rate ranged from 70 to 156 bpm with a mean of 112 ± 29 bpm.

**Figure 3:**
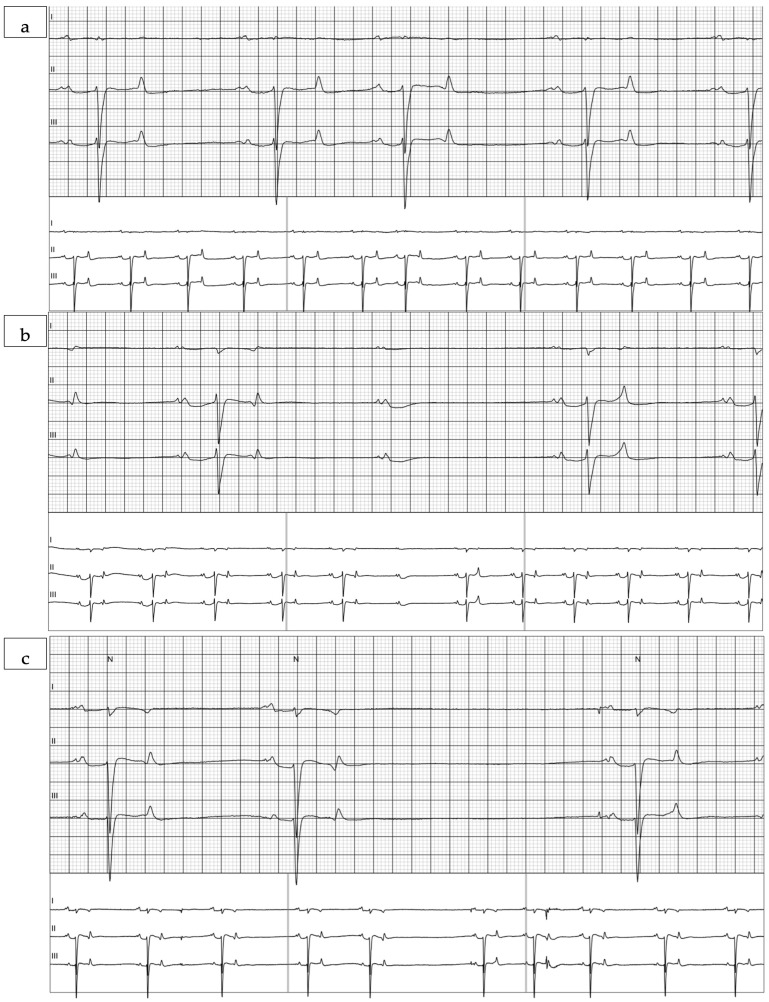
Sample tracings of (**a**) a supraventricular premature complex (SVPC); (**b**) Sample tracing of atrioventricular block; (**c**) Sample tracing of sinoatrial heart block. Tracings are displayed at 25 mm/s paper speed and 10 mm/mV.

**Figure 4:**
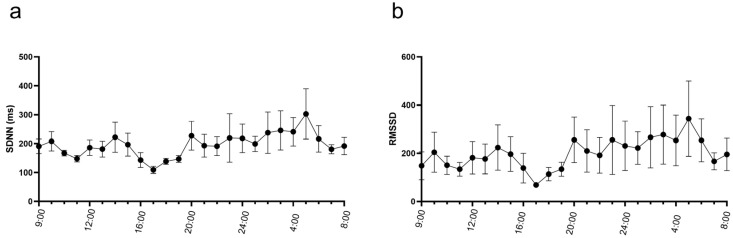
Mean ± standard error of the heart rate variability measures standard deviation of normal intervals (SDNN) (**a**) and root mean square of successive intervals (RMSSD) (**b**) of seven mules calculated hourly during 24 h ambulatory electrocardiograms. Note the decrease in heart rate variability associated with the increase in heart rate in Figure 2.

Rhythm evaluation of the ECGs revealed rare supraventricular premature complexes in five of the seven mules. Individual mules had between 1 and 50 supraventricular complexes in their recording (Figure 3a). No ventricular complexes were noted. Both 2DAVB (Figure 3b) and sinoatrial heart block (Figure 3c) were observed in these recordings. Sinoatrial heart block was observed in two mules, one of which also had atrioventricular block. Atrioventricular block was observed in six of the seven mules. Two mules had a single 2DAVB in the recording. The five mules with more frequent pauses (sinus pauses or 2DAVB) had between 23 and 2945 pauses in their recording, with a median of 202.5 pauses/recording. Heart rate variability values calculated over the 24 h recording are reported in Table 2 and select variables calculated hourly are displayed in Figure 4.

## 4. Discussion

To our knowledge, this is the first report of long-term ambulatory electrocardiographic recordings in mules. The average heart rate for these mules over the recording ranged from 32 to 42 bpm with a mean of 36 ± 3 bpm. This heart rate was similar to the heart rate determined via auscultation, which ranged from 28 to 40 bpm with a mean of 34 ± 5 bpm. While these values are similar to those reported and accepted in horses, they differ from what has previously been auscultated in mules and differ more substantially from values reported via auscultation and aECG in donkeys [8,9,10,11,12,13]. A population of Iberian mules was previously reported to have a higher mean HR of 43.3 bpm [13]. The slightly lower heart rates reported here may be attributed to the nature of the mules in our study, which were performance animals acclimatized to being frequently handled in a training barn environment. These mules may have exhibited a lower stress response and thus experienced a lower heart rate than the less handled Iberian population. By utilizing aECGs over the course of 24 h, we minimized the influence of human interaction during the recording process when compared to auscultation or pulse palpation used in previous mule reports. While this is an advantage of the aECG, it can lead to difficulty in comparison to what may be considered normal via auscultation, as auscultation involves human interaction. As HR can increase in clinical settings, this aspect is an important consideration when comparing our findings with those published. Mules in this study had a lower HR than what is reported in donkeys, with previous studies in donkeys reporting up to 65 bpm on a brief electrocardiogram secured with alligator clips [11,12], or a mean of 48 bpm for donkeys monitored for 24 h [14]. Donkeys have been suggested to have lower vagal tone than horses. Donkeys from these studies are also smaller than the mules studied here, which may have contributed to the higher baseline heart rate. This and the ECG method, which includes handling and restraint, may explain their higher heart rates. The lower heart rates noted in mules in this study suggest that mules, while a hybrid, could exhibit vagal tone more comparable to horses [14]. Future direct comparisons of matched animals under the same conditions are warranted to evaluate the relative contribution of vagal tone, characteristics of the animals themselves, and circumstances of the recordings.

Breed variation is important to consider when interpreting our findings. Our population was limited to Appaloosa (3), Quarter Horse (3), and Warmblood mules (1). As a result, our findings may not be representative of all mules, as different mule breeds may exhibit variation in HR, HRV, and arrhythmia profiles. The small sample size of this study limits the ability to detect variability within a larger population. Our population was an athletic population, which is also a consideration when discussing HR and HRV. Vagal tone is increased in athletic populations, which decreases resting HR and increases HRV. High vagal tone present at rest results in low HRs and benign arrhythmias that disappear when sympathetic tone increases [15,16]. It is possible our population, being performance animals, had higher vagal tone and lower HR than the typical mule seen in clinical practice. Assessing a larger population of mules, of varying breeds, over the course of 24 h could provide additional insight into the vagal tone of the mule and how it compares to that of the horse and the donkey.

The analysis of HRV can provide valuable insight into differences in physiologic and autonomic parameters between mules, horses, and donkeys. Horses have been reported to have higher HRV values when compared to donkeys [17,18,19,20,21]. Utilizing HRV as an indicator of welfare is of particular interest, as mules are commonly used as working animals, and HRV can be collected as a welfare indicator noninvasively. An increase in sympathetic activity of the autonomic nervous system (ANS) results in a reduced HRV. Electrocardiographic recordings that report HRV variables in horses and donkeys over the course of 24 h are lacking. Additionally, comparison of HRV values is a challenge due to a lack of standardized methods. In both horses and donkeys, available HRV data are not reported in a general population to provide reference values but instead are utilized to assess levels of stress and welfare in various populations. Only two prior studies have reported HRV values in the donkey, and none use a 24 h collection period to account for circadian rhythm and variable activity levels within a 24 h period [21,22]. The mean SDNN in our population of mules was 310.7 ± 98.5, which is notably higher than that reported previously in horses and donkeys, indicating a high degree of heart rate variability. RMSSD, an indicator of high-frequency beat-to-beat variations associated with parasympathetic activity, had a median of 108.0 (75.0–327.8) in these mules, which is also higher than reported in donkeys but consistent with that reported in horses over 24 h [21,22,23]. The donkeys used in these prior studies were not only from different populations in different geographical regions, climates, and, in one case, hemispheres, but electrocardiographic data was also collected from a different device. Polar Equine V800 heart rate monitors were utilized in both instances instead of an aECG monitor, which prevents review of the ECG and removal of intervals associated with arrhythmias. These differences in collection methods and population demographics make interpretation difficult; however, our mule population generally exhibits relatively high heart rate variability, which may be consistent with lower stress and higher parasympathetic tone, consistent with the athletic population studied.

When interpreting HRV, it is important to note that HRV is highly activity dependent. Our study did not include accelerometer data or an activity log over the course of the 24 h of recording to compare our hourly HRV data with. Additionally, no direct autonomic measures or standardized stress tests were performed to justify interpretations of lower stress and higher parasympathetic tone.

## 5. Conclusions

Electrocardiographic evaluation of mules over 24 h is feasible, and heart rate parameters were found to be similar to what is described in the horse. Arrhythmias were consistent with the range of cardiac rhythms observed in horses and more frequent than reported in donkeys. Further investigation with an increased population size is warranted to determine if reference values for horses should continue to be used to assess mules, as well as to investigate the prevalence of arrhythmias and their clinical relevance in the mule.

## Figures and Tables

**Table 1:** Mule demographic information (mule number, breed of dam, age, sex, and use).

Mule	Sex	Age	Breed of Dam	Use
Mule 1	Molly	3	Quarter Horse	Show/Pleasure
Mule 2	Gelding	13	Quarter Horse	Endurance
Mule 3	Molly	9	Appaloosa	Show/Pleasure
Mule 4	Molly	10	Appaloosa	Endurance
Mule 5	Gelding	20	Quarter Horse	Gymkhana
Mule 6	Molly	10	Appaloosa	Show/Pleasure
Mule 7	Gelding	9	Warmblood	Show/Pleasure

**Table 2:** Heart rate variability values calculated over the 24 h recording duration for seven mules.

Parameter	Mean ± SD	Median (IQR)	Max	Min	n
RMSSD (ms)	---	109.0 (83.0–243.0)	657.0	51.0	7
SDNN (ms)	317.9 ± 91.9	---	494.0	201.0	7
SDNN Index (ms)	---	161.0 (145.0–201.0)	394.0	129.0	7
SDANN (ms)	214.7 ± 54.8	---	282.0	130.0	7
Triangular Index (ms)	59.0 ± 14.8	---	85.0	42.0	7

## Data Availability

Data are available in DRYAD (To be posted after review).

## Abbreviations

The following abbreviations are used in this manuscript:

ECG	Electrocardiogram
HRV	Heart rate variability
HR	Heart rate
aECG	Ambulatory electrocardiogram
2DAVB	Second-degree atrioventricular block
SVPCs	Supraventricular premature complexes
VCs	Ventricular complexes
SDNN	Standard deviation of normal intervals
SDANN	Standard deviation of the average NN intervals
RMSSD	Root mean square of successive intervals
TI	Triangular index
SD	Standard deviation
IQR	Interquartile range
bpm	Beats per minute
ANS	Autonomic nervous system
